# Facile synthesis of carbon nitride quantum dots as a highly selective and sensitive fluorescent sensor for the tetracycline detection†

**DOI:** 10.1039/d1ra04272f

**Published:** 2021-07-16

**Authors:** Ruining Bai, Heli Sun, Peng Jin, Jingwei Li, Anzhong Peng, Jieli He

**Affiliations:** College of Pharmacy, Dali University Dali 671000 Yunnan P. R. China hejieli@dali.edu.cn +86-872-2257414

## Abstract

Enhanced blue fluorescent carbon nitride quantum dots (g-C_3_N_4_QDs) were synthesized by a simple solvothermal “tailoring” process from bulk g-C_3_N_4_ and analyzed by various characterization methods. The as-obtained g-C_3_N_4_QDs were successfully applied in the determination of tetracycline (TC) with a good linear relationship in the range of 0.23–202.70 μM. The proposed fluorescent sensor shows excellent stability, good repeatability, high selectivity and outstanding sensitivity to TC with a low detection limit of 0.19 μM. The fluorescence quenching mechanism of g-C_3_N_4_QDs with TC was mainly governed by static quenching and the inner filter effect. The method was successfully applied to monitor TC in tap water and milk powder samples.

## Abstract 2

The g-C_3_N_4_QDs were synthesized by a simple solvothermal “tailoring” process from bulk g-C_3_N_4_ which have a “strong quenching” behaviour in the presence of TC. The proposed fluorescent sensor has been successfully applied to detect TC in actual samples.

## Graphical abstract

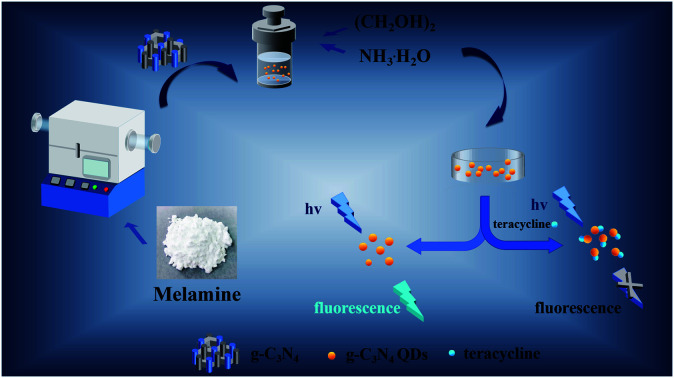

## 1. Introduction

Since its re-discovery in 1990s, graphite carbon nitride (g-C_3_N_4_) as a metal-free semiconductor material has been widely explored and used in sensing,^1–6^ catalysis,^7,8^ fluorescence imaging,^9,10^ and cancer treatment^11^ due to its unique electronic structure, excellent chemical and thermal stability, and good biocompatibility. Usually, g-C_3_N_4_ is prepared by the high-temperature pyrolysis of nitrogen-rich precursors such as melamine, cyanamide, and dicyandiamide.^12^ However, the synthetic bulk g-C_3_N_4_ materials generally have low specific surface area, poor luminescence performance, and insolubility in most solvents, which limit their practical applications. Therefore, it is rewarding to search for solutions to overcome the shortcomings and limitations of bulk g-C_3_N_4_ materials. Quantum dots (QDs) as quasi-zero dimensional nanomaterials have attracted wide attention because of quantum effects, and possess exotic features, such as large specific surface area, good water solubility, and unique optical and electronic properties superior to those of large particles. Currently, researchers have developed various types of QDs including metallic QDs,^13^ nonmetallic QDs,^14^ and composite QDs^15^ among others and applied them in electroluminescence devices, solar cells, photocatalysis, imaging and sensing based on their extraordinary optical properties: electrochemiluminescence, phosphorescence, and fluorescence.^16^ Thus, exploration of g-C_3_N_4_QDs may provide the promising properties and applications of g-C_3_N_4_ materials. As a result, a series of fluorescent g-C_3_N_4_QDs have been fabricated and used as promising fluorescent sensor materials in recent years.^17–19^ For instance, K. Patir *et al.* applied g-C_3_N_4_QDs as a photoluminescent sensor for Hg^2+^ detection.^19^ Up to now, several routes such as hydrothermal, solvothermal, microwave and chemical etching, which are mainly categorized into top-down or bottom-up approaches, have been employed to synthesize QDs.^16,20^ However, the present methods of g-C_3_N_4_QDs whether top-down or bottom-up synthesis are usually complicated and difficult to control. Therefore, developing highly efficient and facile methods to synthesize g-C_3_N_4_QDs is of great importance. In this paper, we report a facile and environmentally friendly solvothermal “tailoring” method to synthesize fluorescent g-C_3_N_4_QDs. Moreover, the application of g-C_3_N_4_QDs still needs further exploration.

Tetracycline (TC) is one of the major broad-spectrum antibiotics, which can inhibit a wide variety of bacteria, and has been extensively used in human therapy, animal disease control and agricultural feed additives because of its excellent therapeutic effect and low cost.^21^ Nevertheless, the absorption and metabolism of TC in the body represent only a small proportion, and about 30 to 90% of TC is released into excreta in the form of parent compounds or metabolites.^22^ Meanwhile, TC commonly has a long halflife in natural environments.^21^ Consequently, the abuses of TC results in residues widely present in animal products, soil, surface water, drinking water and groundwater, which would inhibit aquatic species growth and development.^23^ In addition, TC residues would gradually accumulate in the food chain and finally affect the health of human beings.^23^ Therefore, the rapid and accurate quantitative determination of TC concentration in natural environments is very necessary. Common methods for TC detection include the microbiological method,^24^ high performance liquid chromatography,^25^ enzyme immunoassay,^26^ and capillary electrophoresis.^27^ However, these detection methods have some disadvantages, such as poor detection sensitivity and selectivity, complex sample preparation, tedious operation, costly equipment, being time-consuming and using toxic reagents. Hence, developing a simple, inexpensive, eco-friendly, and rapid method with high selectivity and sensitivity is meaningful. The fluorescent sensing method based on observation of direct emission quenching as the sensing signal is considered to have the advantages of high sensitivity and selectivity, simple operation and repeatability. Therefore, we aim to develop g-C_3_N_4_QDs as an efficient fluorescent sensor to detect TC.

**Scheme 1:**
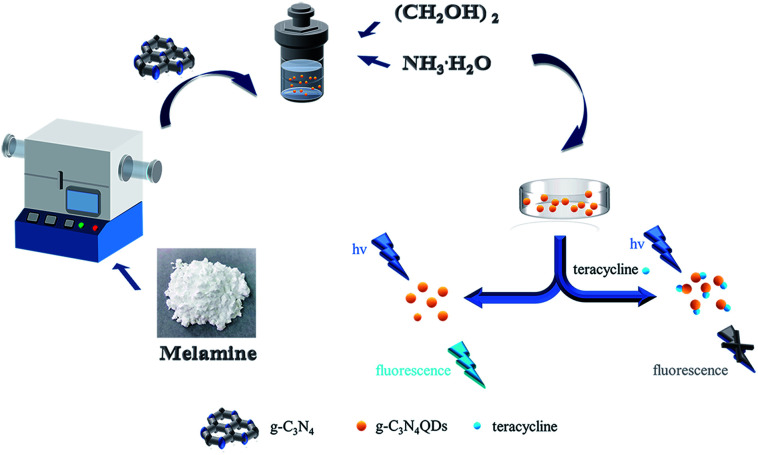
Schematic illustration of the preparation of g-C_3_N_4_QDs and their fluorescence quenching property triggered by tetracycline.

Herein, we succeeded in synthesizing g-C_3_N_4_QDs with low costs, water solubility and bright blue fluorescence *via* a facile and environmentally friendly solvothermal “tailoring” method. The prepared g-C_3_N_4_QD material displayed good stability, reproducibility, high selectivity and sensitivity for TC determination on the basis of the fluorescence quenching method (Scheme 1), and was also successfully applied to detect TC in real water and milk powder samples. Moreover, the fluorescence quenching mechanism of g-C_3_N_4_QDs was proposed.

## 2. Experimental

### 2.1 Reagents and materials

Melamine, ethylene glycol, and ammonia were purchased from Shanghai Sinopharm Chemical Reagent Co., Ltd. TC, chlorotetracycline hydrochloride (CTC), oxytetracycline (OTC), doxycycline hydrochloride (DTC), ciprofloxacin (CIP), amoxicillin (AMX), sulfadiazine (SDZ), and chloramphenicol (CAM) were purchased from Aladdin Industrial Corporation. All reagents were of analytical grade and used as received without further purification.

### 2.2 Preparation of bulk g-C_3_N_4_

Firstly, the bulk g-C_3_N_4_ was synthesized by a simple thermal polymerization of melamine according to our previous work.^28^ Typically, 5 g of melamine was heated to 500 °C in a tube furnace at a ramping rate of 10 °C min^−1^ and kept at this temperature for 2 h and then continued to heat up to 520 °C at the same heating rate and kept for another 2 h. After naturally cooling to room temperature, the obtained yellow product was ground into a homogeneous powder.

### 2.3 Preparation of g-C_3_N_4_QD_S_

The g-C_3_N_4_QDs were prepared by a solvothermal method according to ref. 29. The bulk g-C_3_N_4_ (0.10 g) was mixed with ethylene glycol (15 mL) and ammonia (15 mL). The mixture was transferred into a Teflon-sealed autoclave and kept at 180 °C for 12 h. The resultant product was cooled to room temperature and filtered with a 0.22 μm membrane. Finally, the obtained filtrate containing highly dispersed g-C_3_N_4_QDs was stored at 4 °C before use.

### 2.4 Characterization

The X-ray diffraction (XRD) patterns were obtained with an X-ray diffractometer (Bruker, Germany) using Cu K_α_ radiation (40 kV, 30 mA). A transmission electron microscope JEM-2100F (Japan JEOL Ltd.) was used to record transmission electron micrographs (TEM) at an acceleration voltage of 200 kV. The XPS measurements were carried out on an ESCALAB 250Xi spectrometer (Thermo Scientific, USA) with a pass energy of 30 eV and 100 W. The absolute quantum yield is measured with a C11347-11 Quantaurus-QY absolute quantum yield measurement instrument (Hamamatsu, Japan). Fourier transform infrared spectra (FT-IR) were collected with a Thermo Scientific Nicolet 380 FT-IR spectrometer with a resolution of 4 cm^−1^. The ultraviolet-visible (UV-Vis) absorption spectra were measured on a TU-1901 UV-Vis spectrophotometer (UV). The photoluminescence (PL) spectra were recorded on a Shimazu RF-5301 PC fluorescence spectrophotometer.

### 2.5 Measurement of fluorescence quantum yield

The photoluminescence absolute quantum yield (QY) of the prepared g-C_3_N_4_QDs was measured using an absolute PL quantum yield spectrometer with an integrating sphere (C11347-11, Hamamatsu, Japan) under excitation with a 150 W xenon light source at 325 nm. The test principle and method were referred to ref. 30 and 31. The absolute fluorescence quantum yield = the number of emitted photons/the number of absorbed photons.^30^

### 2.6 Fluorescence detection of TC

0.5 mL of TC solution with various standard concentrations was mixed with 0.5 mL of g-C_3_N_4_QD solution and then diluted with deionized water to 5 mL (the final concentration was 0.3 mg mL^−1^). After being stirred thoroughly and placed for 15 min at room temperature, the fluorescence intensity of the mixture was collected under an excitation wavelength of 325 nm. To explore the selectivity, PL of the solution containing g-C_3_N_4_QDs and other substances (CTC, OTC, DTC, CIP, AMX, SDZ, CAM, Mg^2+^, Ba^2+^, K^+^, *etc.*) was detected using the same method. Moreover, PL intensities of the mixture of g-C_3_N_4_QDs and TC in the presence of other substances were also recorded under identical conditions.

### 2.7 Fluorescence detection of TC in tap water and milk powder samples

The tap water samples were obtained from our lab, filtered through 0.22 μm membrane filters for further analysis and added with a series of different concentration levels of TC. After that, the solutions were analyzed with the same proposed method.

Milk powder samples were obtained from the local supermarket. The procedure for pretreating actual samples was performed on the basis of the reported works with little modification as follows.^32,33^ Firstly, 2 g of milk powder was diluted to 20 mL with ultrapure water. Then 2 mL of 10% trichloroacetic acid (w : v, in water) was added into the sample solution and sonicated for 30 min to precipitate proteins and dissolve other organics in the matrix. After that, the mixture was centrifuged at 8000 rpm for 10 minutes to separate the precipitate. Thereafter, the supernatant was filtered with a 0.22 μm filter membrane to remove lipids, and the filtrate was taken for further analysis.

## 3. Results and discussion

### 3.1 Physicochemical characterization of g-C_3_N_4_QDs

**Fig. 1:**
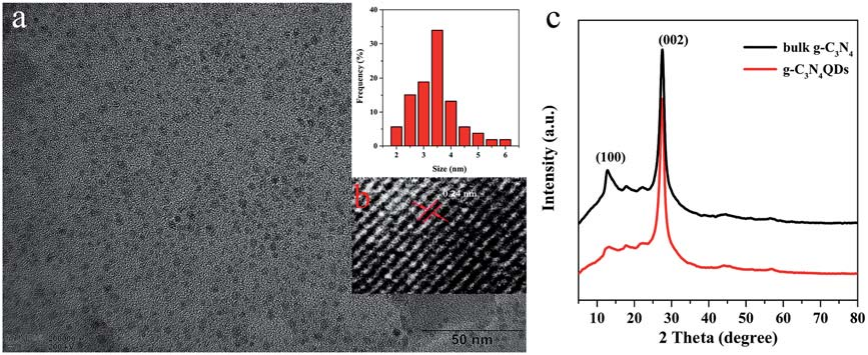
TEM (a), size distribution (a, inset) and HRTEM (b) images of g-C_3_N_4_QDs; XRD patterns (c) of the prepared bulk g-C_3_N_4_ and g-C_3_N_4_QDs.

TEM and high resolution TEM were employed to observe the morphology of g-C_3_N_4_QDs. As presented in Fig. 1a, the g-C_3_N_4_QDs are well mono-dispersed and have a relatively uniform spherical shape. The particle sizes are mostly in the range of 2–6 nm with a narrow distribution, and the average particle size is approximately 3.5 nm. Fig. 1b shows the HRTEM images of g-C_3_N_4_QDs, and the spacing of the lattice fringe is 0.24 nm corresponding to the (100) plane of g-C_3_N_4_.^34^Fig. 1c displays the XRD patterns of bulk g-C_3_N_4_ and g-C_3_N_4_QDs. As shown, the diffraction peaks of g-C_3_N_4_QDs are in good agreement with those of bulk g-C_3_N_4_, indicating that they have the same basic crystal structure. The strong peak at 27.5° is corresponding to the (002) plane, and a relatively weak diffraction peak at 13.1° is attributed to the (100) plane. These two characteristic diffraction peaks reflect the inter-planar stacking of the aromatic ring structure and the in-planar tri-*s*-triazine unit packing motif, respectively.^35^ Moreover, compared with bulk g-C_3_N_4_, the relative intensity of the (100) peak for g-C_3_N_4_QDs is much weaker, indicating that the in plane repeated unit structure may be damaged to a certain extent after solvothermal treatment.^36^

**Fig. 2:**
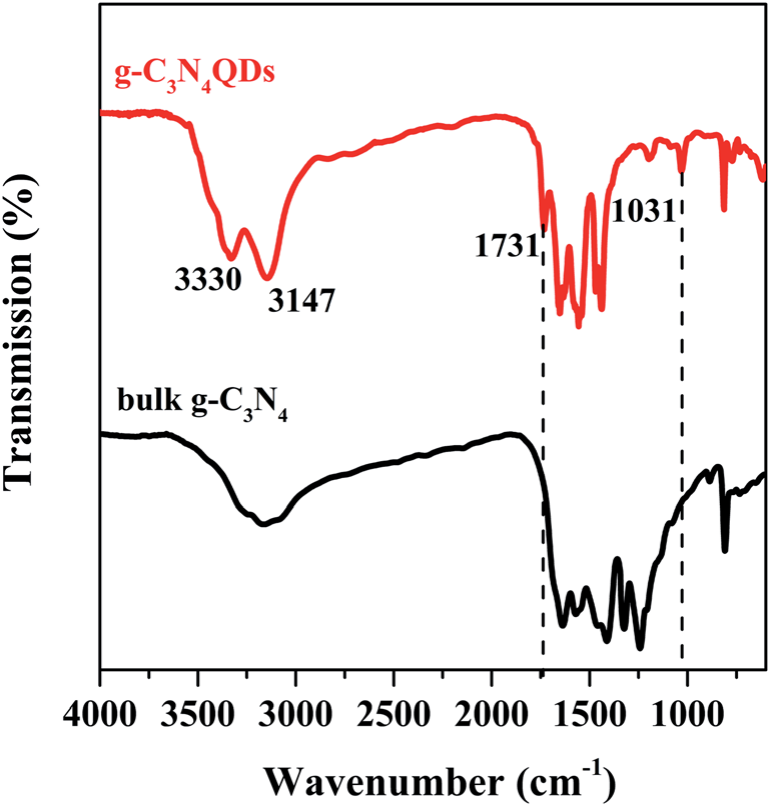
FT-IR spectra of g-C_3_N_4_ and g-C_3_N_4_QDs.

The FT-IR spectra of bulk g-C_3_N_4_ and g-C_3_N_4_QDs are presented in Fig. 2. For both samples, they have an absorption peak located at 810 cm^−1^ belonging to the out of plane breathing vibration modes of heptazine heterocyclic rings.^37,38^ Several intense bands in the range of 1200–1650 cm^−1^ are also detected for both bulk g-C_3_N_4_ and g-C_3_N_4_QDs, which are attributed to typical stretching vibrations of tri-*s*-triazine units. The results indicated that the basic surface functional structures of g-C_3_N_4_ are not changed in the process of solvothermal “tailoring”. Moreover, the broad bands in the 3000–3500 cm^−1^ region assigned to stretching vibrations of N–H (−3100 cm^−1^) and O–H (−3300 cm^−1^) of adsorbed water are observed for the two samples.^11^ Nevertheless, the intensity of the N–H and O–H peaks for g-C_3_N_4_QDs is much stronger than that of g-C_3_N_4_. And these hydrophilic groups are probably beneficial to increase the water solubility of g-C_3_N_4_QDs. In addition, compared with bulk g-C_3_N_4_, two new peaks at *ca.* 1731 and 1032 cm^−1^ appeared, which are ascribed to C=O stretching vibration and C–O vibration in the C–O–C group, respectively.^39,40^ It seems possible that oxygen atoms may be introduced into g-C_3_N_4_QDs.

**Fig. 3:**
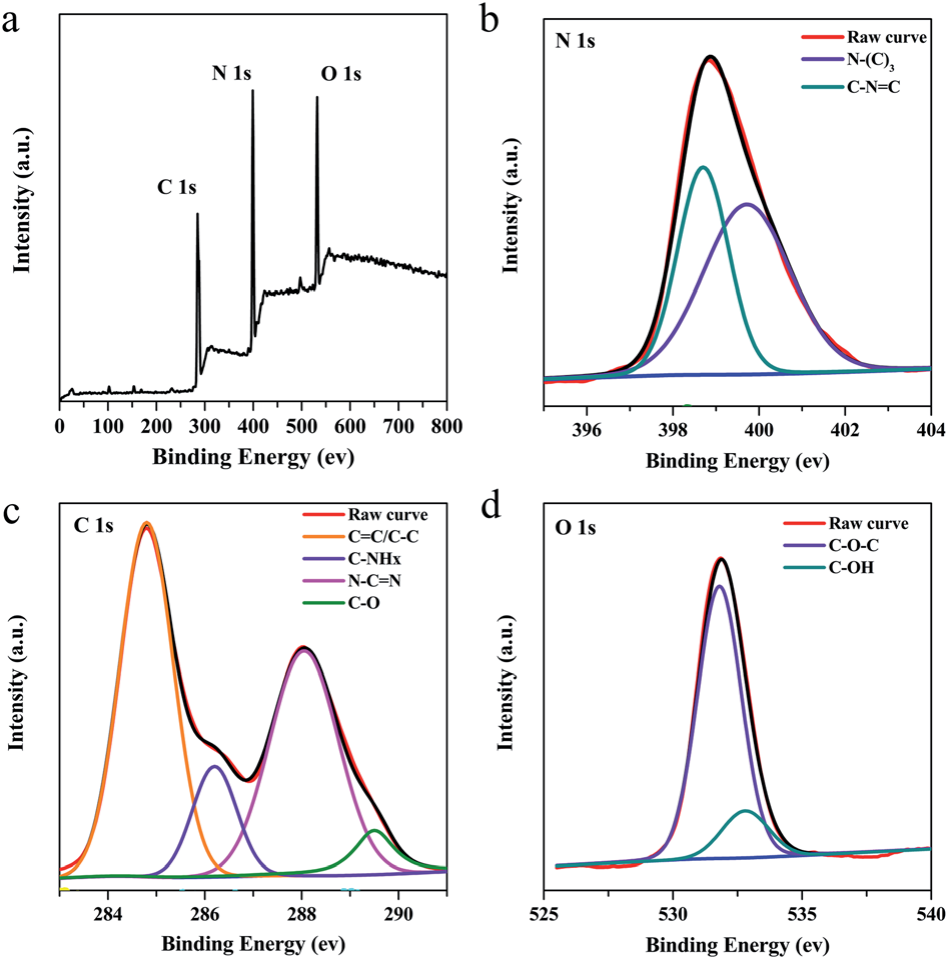
XPS spectra of g-C_3_N_4_QDs: (a) survey spectra, (b) N 1s, (c) C 1s and (d) O 1s.

XPS was performed to further investigate the chemical composition and structure information of g-C_3_N_4_QDs. The survey scan of the XPS spectrum displays three peaks located at 284.8, 398.7 and 532.8 eV (Fig. 3a), which are ascribed to C 1s, N 1s, and O 1s, correspondingly. In the high resolution N 1s spectrum (Fig. 3b), g-C_3_N_4_QDs possess two types of nitrogen species: N–(C)_3_ (399.7 eV) and C–N=C (398.7 eV).^1,41^ The deconvolution of the C 1s spectrum (Fig. 3c) presents four peaks at 284.8, 286.3, 288.1, and 289.5 eV, which could be ascribed to graphitic carbon (sp^2^ C=C or sp^3^ C–C), C–NH_*x*_ (*x* = 1, 2), N–C=N, and C–O.^12,42–44^ For the O 1s spectrum (Fig. 3d), it can be deconvoluted into two peaks at 531.8 eV and 532.8 eV, which are related to C–O–C and hydroxyls of adsorbed water, respectively.^45,46^ The XPS results further confirm the presence of oxygen impurities in the g-C_3_N_4_QDs, which may promote the formation of intermolecular hydrogen bonds and thus enhance water solubility.

### 3.2 Optical properties of g-C_3_N_4_QDs

**Fig. 4:**
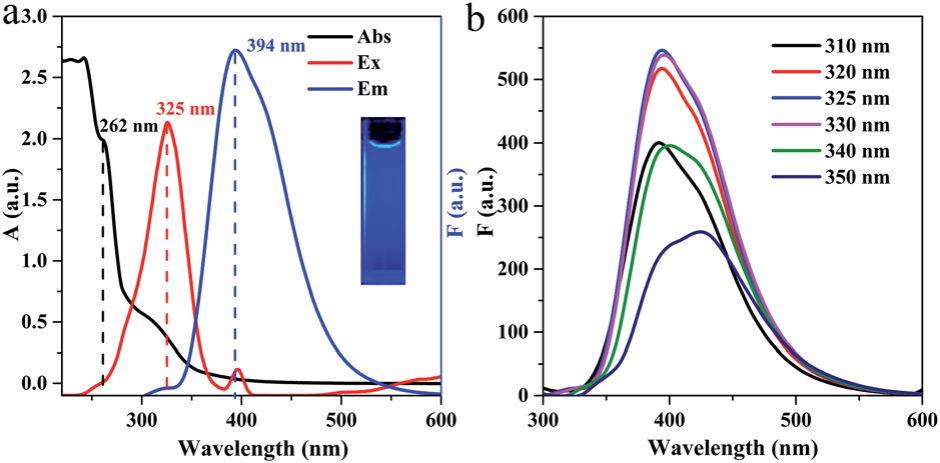
UV-vis absorption and fluorescence spectra (a) and fluorescence emission spectra at different excitation wavelengths (b) of g-C_3_N_4_QDs. Inset of (a): photograph taken under 325 nm UV light.

Fig. 4a shows the UV-Vis absorption and fluorescence spectra of g-C_3_N_4_QD solution. The large peak from 200 to 270 nm with a shoulder centered at 262 nm is from π–π* electronic transition of the tri-*s*-triazine ring in graphitic carbon nitride.^47,48^ Furthermore, another broad peak appears between 300 and 340 nm because of n–π* electronic transition of the C–N bond in the heptazine heterocycle structure.^45^ The excitation spectrum exhibits a maximum peak at 325 nm, and an emission peak emerges at 394 nm when g-C_3_N_4_QDs are excited at this maximum excitation wavelength. Moreover, the g-C_3_N_4_QD solution shows bright blue fluorescence under irradiation with 325 nm UV light (Fig. 4a inset). Compared with our previous as-synthesized few-layer g-C_3_N_4_ (ref. 28) and much of g-C_3_N_4_ materials,^49,50^ the absorption and fluorescent behaviors of the as-prepared g-C_3_N_4_QDs show a clear blue shift, which could be due to the quantum confinement effect.^43^ The absolute quantum yield of the obtained g-C_3_N_4_QDs is 0.097, which is slightly higher than that of the reported carbon dots.^51–53^Fig. 4b displays the emission spectra of g-C_3_N_4_QDs at various excitation wavelengths. Commonly, surface defects can act as a capture center for excitons, resulting in surface defect state fluorescence. Surface defect fluorescence is caused by radiation relaxation from the excited state to the ground state, which can lead to multicolor emissions. When the sample is excited by light with different specific wavelengths, the photons whose energy satisfies the optical band gap will transit and accumulate in the adjacent surface defect centers, and then return to the ground state to emit different wavelengths of light, therefore showing excitation wavelength-dependent characteristics.^54,55^ As shown in Fig. 4b, with excitation wavelength shifting from 310 to 330 nm, the emission peak positions are almost not changed. With continuously increasing the excitation wavelength from 330 to 350 nm, the emission peaks become broadened and appear slightly red-shifted. The maximum fluorescence emission peak is obtained when excited at 325 nm. This emission behavior suggests that there would be a slight inhomogeneity of particle size and a few surface defects involved in the surface functional groups of g-C_3_N_4_QDs, which therefore result in nonuniform surface states, which is consistent with the results of FT-IR and XPS.

### 3.3 Sensing for TC

**Fig. 5:**
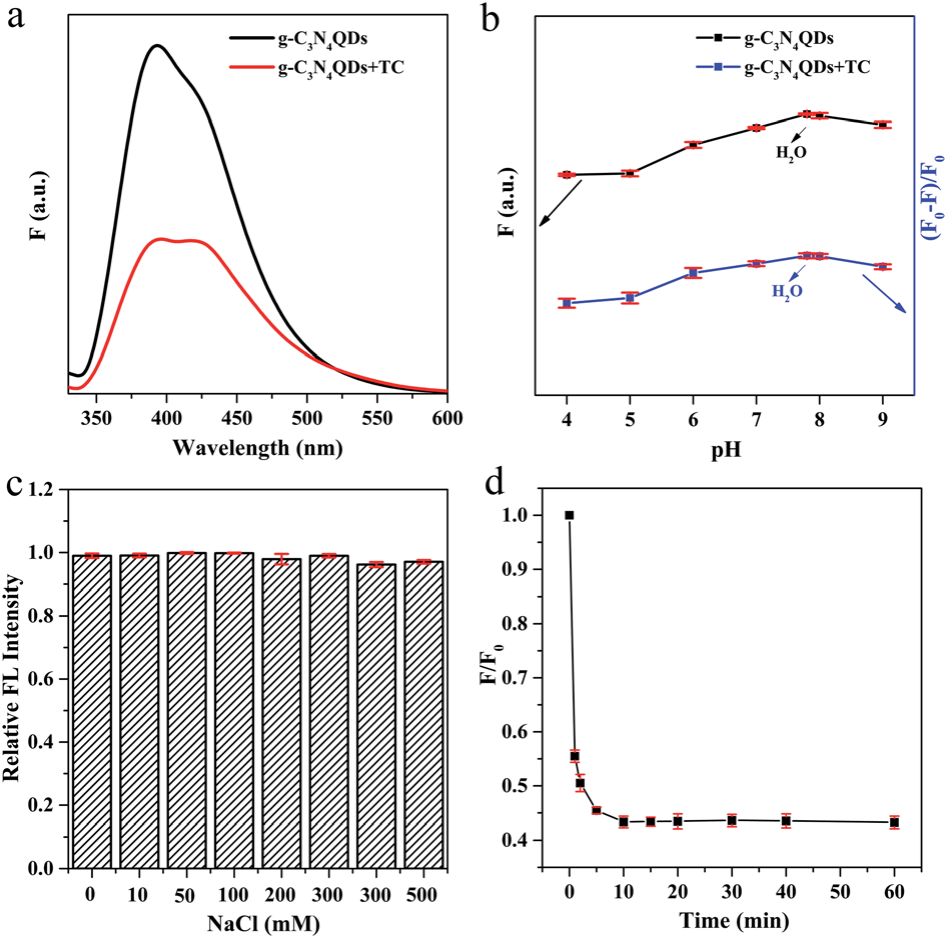
Fluorescence spectra of g-C_3_N_4_QDs without and with TC (a), and pH- (b), salt concentration- (c) and time-dependent (d) fluorescence response of g-C_3_N_4_QDs with TC.

To evaluate the potential application of g-C_3_N_4_QDs as an optical sensor, we investigated the g-C_3_N_4_QDs as a fluorescent probe to detect TC. As shown in Fig. 5a, the fluorescence intensity decreases sharply when TC is added into the g-C_3_N_4_QD solution, indicating that its fluorescence is sensitive to TC. Moreover, the effect of pH on the fluorescence intensity was explored. *F*_0_ and *F* represent the PL intensities of g-C_3_N_4_QDs without and with TC. As depicted in Fig. 5b, with the pH value changing from 4.0 to 9.0, the PL intensity of g-C_3_N_4_QDs gradually increases first and reaches a maximum under neutral conditions (pH 7–8), and then decreases slowly. In the presence of TC, the quenching efficiency [(*F*_0_ − *F*)/*F*_0_] displays almost the same change trend. In view of that the neutral ultrapure water system is environmentally friendly and readily available, using the ultrapure water (pH 7–8) as the solvent is also researched, and the results showed that the fluorescence intensity and quenching efficiency can reach the maximum in the ultrapure water system. Further studies reveal that the PL intensity can remain unaffected by ion strength when adding NaCl into the ultrapure water system with the salt concentration increasing from 0 to 500 mM (Fig. 5c). Therefore, ultrapure water is selected as a solvent in the following research. In addition, the PL intensity drops quickly with the addition of TC in the first 10 min and then reaches balance within 15 minutes (Fig. 5d), implying that the fluorescence quenching of g-C_3_N_4_QDs is quite rapid and can be applied in fast sensing of TC. What's more, the PL intensity can remain stable during the following continuous irradiation for 60 minutes, which verifies the good light stability of the as-prepared g-C_3_N_4_QDs. Hence, the reaction time between TC and g-C_3_N_4_QDs is determined to be 15 min.

**Fig. 6:**
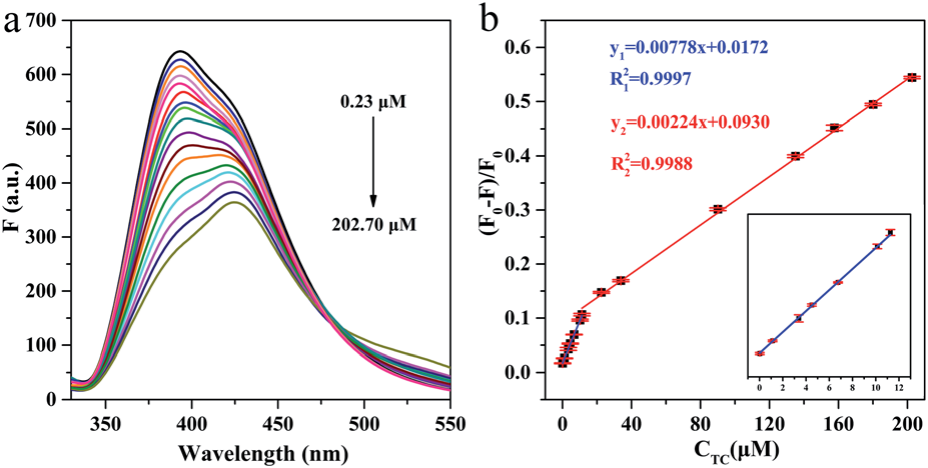
Fluorescence emission spectra of g-C_3_N_4_QDs with different concentrations of TC (a) and the relationship between the fluorescence quenching efficiency and the concentration of TC (b).

Under the optimized experimental conditions, the analytical performance of g-C_3_N_4_QDs towards different concentrations of TC was evaluated. As shown in Fig. 6a, the fluorescence intensity of g-C_3_N_4_QDs is gradually declined with the increase of TC concentration. As a note, the signals exhibit drift which often occurs in optical chemical sensors because of scattering, photo-bleaching and so on. Fluorescence quenching efficiency *vs.* TC concentration exhibits good linear relationships in the range of 0.23 to 11.26 μM and 11.26 to 202.70 μM (Fig. 6b). The corresponding calibration equations are *y* = 0.00778*x* + 0.0172 (*R*^2^ = 0.9997) and *y* = 0.00224*x* + 0.0930 (*R*^2^ = 0.9988), where *y* and *x* are the quenching efficiency [(*F*_0_ − *F*)/*F*_0_] and the concentration of TC, respectively. The limit of detection (LOD) of 0.19 μM is calculated according to 3 times the standard deviation of the blank measurements divided by the slope of the standard curve (LOD = 3SD/*s*). For comparison, some of the reported fluorescence sensors based on other materials for TC detection are displayed in Table 1, indicating that the proposed sensing system has superior sensitivity, a wider detection range and a relatively lower detection limit.

**Table 1:** Comparison of fluorescence detection methods based on different materials for the measurement of TC

Probes	Linear range (μM)	LOD (μM)	Ref.
Carbon dots	10.0–400	6.0	56
Europium-doped carbon quantum dots	0.5–200	0.3	57
A30 DNA-templated AuNCs	0.1–60	0.02	32
Nitrogen and sulfur co-doped carbon dots	0.369–73.7	0.148	58
Carbon dots	0.5–6	0.33	59
CdS quantum dots	15–600	7.78	60
g-C_3_N_4_QD_S_	0.23–202.70	0.19	This work

**Fig. 7:**
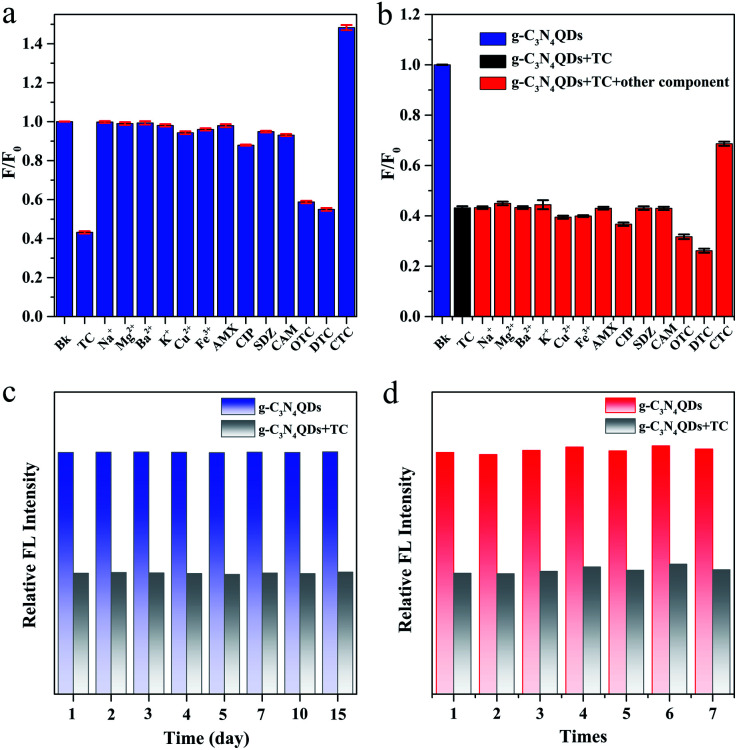
The relative fluorescence intensity of g-C_3_N_4_QDs with TC (200 μM) or various ions and antibiotics (400 μM) (a), and with mixtures of TC (200 μM) and other ions and antibiotics (400 μM) (b), and effects of storage time (c) and preparation times (d) on the fluorescence intensity of g-C_3_N_4_QDs without and with TC.

The selectivity of the present method was further investigated in the presence of potentially interfering representative metal ions (Na^+^, Mg^2+^, Ba^2+^, K^+^, Cu^2+^, and Fe^3+^) and common antibiotics (AMX, CIP, SDZ, CAM, TC, CTC, OTC, and DTC). As described in Fig. 7a, the fluorescence of g-C_3_N_4_QDs is distinctly quenched only by TC, OTC and DTC, which belong to tetracycline antibiotics (TCs). Among them, the quenched efficiency of TC is the highest. However, another tetracycline antibiotic CTC produces a fluorescence enhancement phenomenon. What's more, except TCs, other above mentioned components show negligible fluorescent quenching. Moreover, the experiments of adding the mixtures of 200 μM TC and 400 μM of the above mentioned components were performed. The interference effects of other coexisting metal ions and antibiotics except TCs on the fluorescence quenching of TC can be ignored (Fig. 7b). Therefore, it can be concluded that the fluorescent sensor based on the as-synthesized g-C_3_N_4_QDs has excellent selectivity to TCs.

The stability of g-C_3_N_4_QDs was also studied. The relative fluorescence intensities of g-C_3_N_4_QDs in the absence and presence of TC are basically unchanged after storage for 15 days at room temperature (Fig. 7c), which demonstrates that the as-obtained fluorescent sensor of g-C_3_N_4_QDs has excellent stability. Additionally, to evaluate the reproducibility of g-C_3_N_4_QDs, we synthesized g-C_3_N_4_QDs repeatedly 7 times. The relative fluorescence intensities of g-C_3_N_4_QDs with and without TC remain almost constant (Fig. 7d). The relative standard deviation (RSD) of TC detected for the seven parallel tests is 1.10%, and the average recovery calculated as average measured value/spiked value × 100% is 99.63% (Table S1†), indicating that the presented g-C_3_N_4_QD fluorescent sensor is quite satisfactory in reproducibility.

### 3.4 Statistical evaluation

Statistical evaluation was employed to check the feasibility of the proposed fluorescence sensor method for TC detection. TC standard solutions of 9.01 and 157.66 μM were prepared and determined by the presented method in six parallel tests. The measured values, mean values and standard deviations (*S*) are summarized in Table S2.† A 95% confidence level was selected in the statistical evaluation. Considering that the outliers may affect the accuracy and precision of the results, the Grubbs test was used to determine whether outliers should be discarded.^61^ The *G* value was calculated by formula (1).1
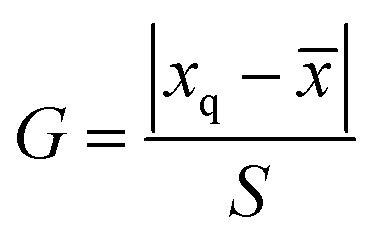
2
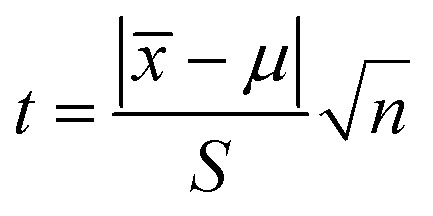
where *x*_q_ is the questionable value and *x̄* is the average value. As shown in Table S2,† the outliers of the two sets of data are 9.31 and 158.22 μM, respectively. By calculation, the *G* values of the two sets are 1.28 and 1.24, which are both lower than the critical value *G*_0.05,6_ of 1.89.^62^ Therefore, the outliers of 9.31 and 158.22 μM should be retained. After checking the outliers, the *t* test was used to determine the systematic error of the test data. Formula (2) was used to calculate the value of *t*. If *t* ≥ *t*_*α*,*f*_, where *α* = 1 − 0.95 = 0.05 and *f* = *n* − 1, it means that there is a significant difference between the average measured value and true value. Otherwise, there is no significant difference. The calculated *t* values of the two sets of data are 0.59 and 2.24 respectively, which are lower than the *t*_0.05,5_ of 2.571.^62^ Hence, there is no significant difference between the average measured value and true value. Moreover, the RSDs of the two sets of data are 3.30% and 1.10%, respectively, indicating that the precision is satisfactory. Above all, the described fluorescence sensor method is feasible for TC detection within the range of the two calibration curves with high accuracy and precision.

### 3.5 Application in actual samples

Based on the above results, the standard addition method was conducted to examine the practical application of the proposed fluorescent sensor in tap water and milk powder samples. The concentration monitored in all samples was derived from the standard curves and regression equations. As described in Table S3,† recoveries of TC at the four fortification levels in assay in tap water and milk powder were determined to be 97.19–102.76% with RSDs of 0.23–3.75%, and 95.08–104.85% with RSDs of 0.41–3.14%, correspondingly. The results indicated that the proposed fluorescent probe is reliable and can be applied to detect TC in real samples.

### 3.6 Proposed quenching mechanism

We further researched the fluorescence quenching mechanism of g-C_3_N_4_QDs caused by TC. Fluorescence quenching is divided into static and dynamic quenching processes.^63^ The static quenching is due to the formation of a non-luminescent complex between the ground state fluorescent substance and quencher.^63^ The collisional interaction between the quencher and excited state fluorescent substance will result in dynamic quenching.^63^ The dynamic quenching can be described by the Stern–Volmer equation [Eqn (3)], and the static quenching process has a similar equation form [Eqn (4)].

3 *F*_0_/*F* = 1 + *K*_sv_[Q]

**Fig. 8:**
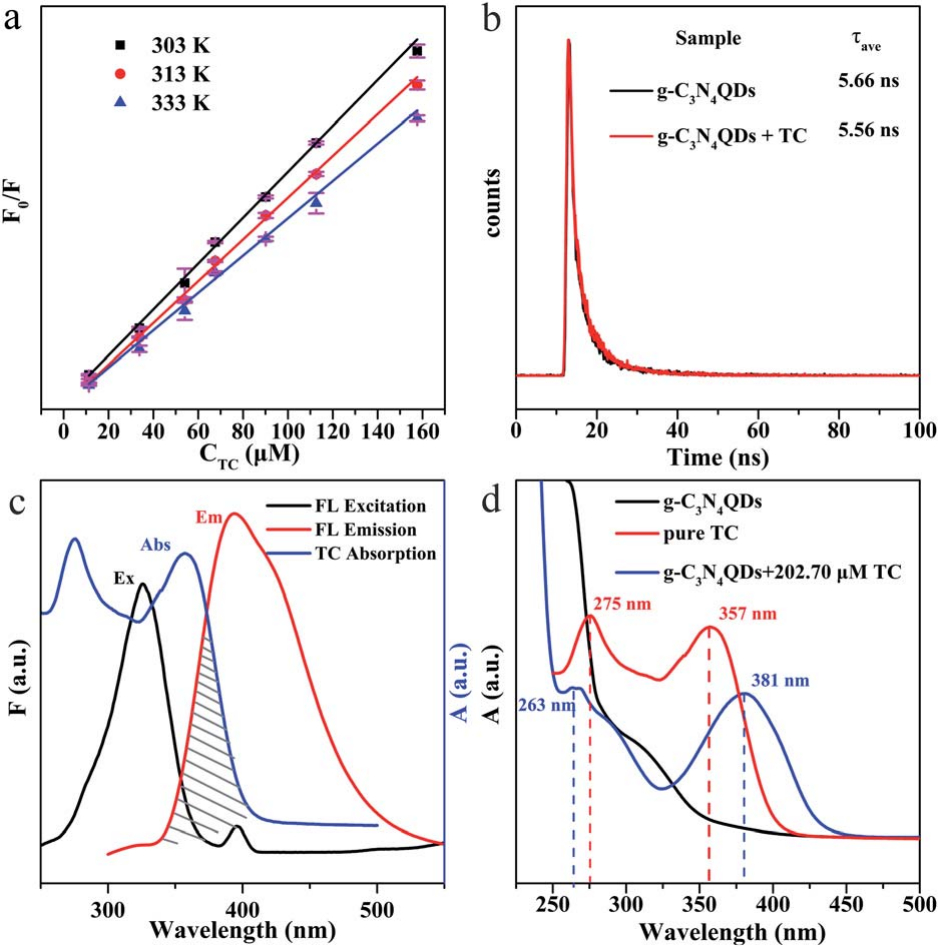
(a) Stern–Volmer plots of g-C_3_N_4_QDs with TC at different temperatures; (b) fluorescence decay curves of g-C_3_N_4_QDs with and without TC; (c) fluorescence excitation and emission spectra of g-C_3_N_4_QDs and UV-vis absorption spectra of TC; (d) UV-vis absorption spectra of g-C_3_N_4_QDs, TC and g-C_3_N_4_QDs-TC.

4 *F*_0_/*F* = 1 + *K*_s_[Q]

where *F*_0_ and *F* are the fluorescence intensities of g-C_3_N_4_QDs in the absence and presence of TC, respectively; *K*_sv_ represents the quenching constant; *K*_s_ denotes the association constant of the complex; [Q] is the concentration of the quencher. The curves of *F*_0_/*F vs.* [Q] at various experimental temperatures are shown in Fig. 8a, and the parameters of plots are displayed in Table S4.† The slopes of linear regression equations are 4.56 × 10 ^3^, 4.15 × 10 ^3^ and 3.71 × 10 ^3^ L mol^−1^ at the temperature of 303, 313 and 333 K, correspondingly. Obviously, the quenching efficiency is reduced with the increase of temperature, indicating that there is a static quenching process,^64^ because of that increasing the temperature will decrease the association constant of the complex formed between g-C_3_N_4_QDs and TC.

In addition, we also measured the fluorescence lifetime of g-C_3_N_4_QDs without and with TC (Fig. 8b), and the fluorescence decay curves are well-fitted by a three-exponential function [Eqn (5)]. Among them, *τ*_1_, *τ*_2_ and *τ*_3_ are the time constants of the three radiative decay channels; *A*_1_, *A*_2_ and *A*_3_ are the corresponding amplitudes.^65^ The relevant parameters are summarized in Table S5,† and the calculated average lifetimes are 5.66 and 5.56 ns for g-C_3_N_4_QDs and g-C_3_N_4_QDs/TC based on eqn (6), respectively. Nearly unchanged fluorescence lifetime further indicates that the fluorescence quenching of g-C_3_N_4_QDs with TC addition is mainly governed by a static quenching process.5
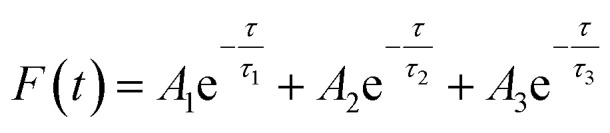
6
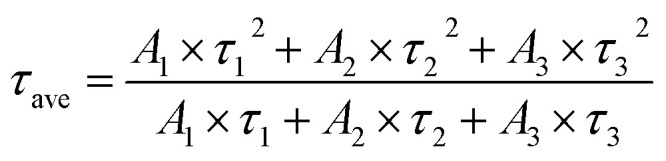


Furthermore, in order to get more information about the mechanism of fluorescence quenching, we analyze the fluorescence spectra of g-C_3_N_4_QDs and the UV-vis absorption spectrum of TC carefully (Fig. 8c). The excitation and emission spectra of g-C_3_N_4_QDs overlap with the absorption peaks of TC, which suggests that the fluorescence of g-C_3_N_4_QDs may be quenched either by the inner filter effect (IFE) or fluorescence resonance energy transfer (FRET). FRET is a non-radiative energy transfer caused by vibration collision within a limited distance of 10 nm between the donor and acceptor, which will shorten the fluorescence lifetime of the donor,^66^ whereas the IFE is a radiation energy transfer and not limited by distance, which is induced by the formation of the ground-state complex. Therefore, for the IFE, the absorption spectra change and the fluorescence lifetime remains constant of the fluorescent substance with the quencher.^65^ As shown in Fig. 8d, the absorption peaks of TC shift from 275 and 357 nm to 263 and 381 nm accordingly, and the relative intensity changes after reaction with g-C_3_N_4_QDs. Moreover, combined with the front results of fluorescence lifetime analysis, we deduced that the fluorescence quenching of g-C_3_N_4_QDs with TC is attributed to the IFE, not FRET.

## 4. Conclusion

In summary, we have successfully prepared a g-C_3_N_4_QD solution with excellent fluorescence properties and water-solubility by a simple, low cost and environmental-friendly solvothermal “tailoring” method. The resultant g-C_3_N_4_QDs have a “strong quenching” behavior in the presence of TC, which is mainly controlled by a static quenching process because of the IFE mechanism. Based on this, we developed a rapid fluorescent sensor to detect TC in a highly selective and sensitive manner with a wide detection range and low detection limit. The proposed g-C_3_N_4_QDs have promising application prospects in the monitoring of TC in real samples.

## Conflicts of interest

There are no conflicts to declare.

## Supplementary Material

RA-011-D1RA04272F-s001
