# Supplementary material for: Facile synthesis of carbon nitride quantum dots as a highly selective and sensitive fluorescent sensor for the tetracycline detection
Source: RSC Adv. 2021 Jul 16;11(40):24892–9. doi: 10.1039/d1ra04272f (PMC9036896; doi:10.1039/d1ra04272f)
Supplement: RA-011-D1RA04272F-s001 [file RA-011-D1RA04272F-s001.pdf]

Electronic Supplementary Material (ESI) for RSC Advances.
This journal is © The Royal Society of Chemistry 2021

Supporting Information

Facile synthesis of carbon nitride quantum dots as a highly selective and sensitive fluorescent sensor for tetracycline detection

*Ruining Bai, Heli Sun, Peng Jin, Jingwei Li, Anzhong Peng, Jieli He**

College of Pharmacy, Dali University, Dali 671000, Yunnan, P. R. China

Table S1 Detailed values of reproducibility experiments.

F_0 (a.u.)	F (a.u.)	Detection value (μM)	Average (μM)	Average recovery (%)	RSD (%, n=7)
618.802	282.066	201.42			
630.713	286.037	202.45			
613.319	284.530	197.80			
593.324	276.509	196.86	199.26	99.63	1.10
616.117	285.617	197.96			
606.573	277.586	200.61			
621.127	288.284	197.71			

(F_0 is the fluorescence intensity of g-C_3N_4QDs without TC; F is the fluorescence intensity of g-C_3N_4QDs with 200 μM TC).

Table S2 Statistical evaluation.

Standard values (μM)	Measured values (μM)	Mean values (μM)	S	RSD (%, *n* = 6)	Outliers (μM)
9.01	9.31	8.94	0.29	0.03	9.31
	8.61				
	9.27				
	8.66				
	8.92				
	8.88				
157.66	156.65	156.09	1.71	0.01	158.22
	154.54				
	157.86				
	154.48				
	158.22				
	154.78				

Table S3 Detection of TC in tap water and milk powder samples.

Samples	Added (μM)	Detected (μM)	Recovery (%)	RSD (%, *n* = 3)
Tap water	6.75	6.80	100.74	2.27
	18.02	18.81	101.22	2.87
	56.31	55.63	97.19	3.75
	157.66	162.01	102.76	0.23
Milk powder	6.75	6.49	96.16	1.17
	18.02	18.89	104.85	3.00
	56.31	57.83	102.69	3.14
	157.66	153.16	95.08	0.41

Table S4 Some parameters of Stern-Volmer plots.

Temperature (T/K)	Stern-Volmer equation (*C*, μM)	Correlation coefficient (R^2)	K_s (L• mol^{-1})
303	$F_0/F = 1.01555 + 4.56\times10^{-3}$ [Q]	0.99836	4.56×10^3
313	$F_0/F = 1.00511 + 4.15\times10^{-3}$ [Q]	0.99688	4.15×10^3
333	$F_0/F = 1.00821 + 3.71\times10^{-3}$ [Q]	0.99687	3.71×10^3

Table S5 Some parameters of fluorescent decay curves.

Sample	τ_1	A_1	τ_2	A_2	τ_3	A_3	τ_{ave}
g-C_3N_4QDs	5.04	285.18	1.48	759.53	19.08	21.79	5.66
g-C_3N_4QDs-TC	1.50	706.64	5.42	347.79	22.54	11.90	5.56
